# Effects of Biochar Addition on CO_2_ and N_2_O Emissions following Fertilizer Application to a Cultivated Grassland Soil

**DOI:** 10.1371/journal.pone.0126841

**Published:** 2015-05-28

**Authors:** Jingjing Chen, Hyunjin Kim, Gayoung Yoo

**Affiliations:** Center for environmental studies, Department of Applied Environmental Science, College of Engineering, Kyung Hee University, 1 Seochon dong, Giheung gu, Gyeonggido, Korea; USDA-ARS, UNITED STATES

## Abstract

Carbon (C) sequestration potential of biochar should be considered together with emission of greenhouse gases when applied to soils. In this study, we investigated CO_2_ and N_2_O emissions following the application of rice husk biochars to cultivated grassland soils and related gas emissions tos oil C and nitrogen (N) dynamics. Treatments included biochar addition (CHAR, NO CHAR) and amendment (COMPOST, UREA, NO FERT). The biochar application rate was 0.3% by weight. The temporal pattern of CO_2_ emissions differed according to biochar addition and amendments. CO_2_ emissions from the COMPOST soils were significantly higher than those from the UREA and NO FERT soils and less CO_2_ emission was observed when biochar and compost were applied together during the summer. Overall N_2_O emission was significantly influenced by the interaction between biochar and amendments. In UREA soil, biochar addition increased N_2_O emission by 49% compared to the control, while in the COMPOST and NO FERT soils, biochar did not have an effect on N_2_O emission. Two possible mechanisms were proposed to explain the higher N_2_O emissions upon biochar addition to UREA soil than other soils. Labile C in the biochar may have stimulated microbial N mineralization in the C-limited soil used in our study, resulting in an increase in N_2_O emission. Biochar may also have provided the soil with the ability to retain mineral N, leading to increased N_2_O emission. The overall results imply that biochar addition can increase C sequestration when applied together with compost, and might stimulate N_2_O emission when applied to soil amended with urea.

## Introduction

Biochar application to agricultural soils is a promising management practice that has the potential to mitigate climate change and increase soil quality [1–5]. However, it is not yet widely used in agricultural fields as a common practice, because the effects of biochar appear to be dependent on the characteristics of the soil and the biochar [6–9].

If biochar is a completely inert material that does not interact with soil components, only the C sequestration potential of biochar needs to be considered, and changes in the physical, chemical, and biological properties of the soil upon biochar addition do not need to be considered. However, biochar is not completely inert, and some portions of biochar, especially the surface, contain significant amounts of bioavailable nutrients [10–12]. Therefore, the addition of biochar to soils can affect the physical, chemical, and biological aspects of the soil, thereby influencing C and N cycles in the soil [10–14].

Changes in soil C and N dynamics will result in changes in CO_2_ and N_2_O emissions. Soil CO_2_ emissions have been reported to increase [15–17], decrease [6, 9, 18], and remain unchanged [19] by biochar amendment. These widely varying observations can largely be explained by different amounts of volatile organic matter content in the biochar, which generally increases with decreasing pyrolysis temperature. Volatile matter content is widely used as an indicator of the amount of labile C in biochar [20, 21]. Changes in CO_2_ emission are also related to the application rate of biochar. Cumulative CO_2_ production was reported to be significantly higher than the control at 1% and 2% biochar application rates [22,23], while there was no change in CO_2_ emission from soil with 5% and 10% biochar application rates. Effect of biochar also depended on the condition of the soil to which it was applied; addition of biochar to soil with a high C content did not result in any additional change in CO_2_ emission [15].

Effects of biochar addition on N_2_O emission are even more inconsistent, because the process of N_2_O emission is very complicated, involving denitrification, autotrophic nitrification, and heterotrophic nitrification, among other processes [24,25].N_2_O emissions are widely known to be influenced by soil water status, available C content, oxygen content, pH, N availability, and so on [26–28]. Reduced N_2_O evolution was reported from rice paddy soil amended with biochar when the soil was relatively wet, while the opposite trend was observed when the soil was drier [29]. A reduction in N_2_O emission by biochar amendment was also reported by [30]. These observations have been explained by enhanced soil aeration [31], increased pH [27], and microbial immobilization of soil NO_3_^-^ by biochar addition [22]. Biochar has also been reported to have opposite effects on N_2_O emission. Higher N_2_O emission was observed from rice paddy soil amended with biochar made from swine manure by [32–34]; the changes in N_2_O emission were attributed to the size of the available inorganic N pool [32–34].

In this study, we monitored changes in CO_2_ and N_2_O emissions from cultivated grassland soil in response to biochar amendment. Although the area of cultivated grassland in South Korea is not large, the government plans to extend this area because of increased demand for domestic forage. Grassland in Korea is also important because soil C storage in this ecosystem ranges from 3847gC m^-2^ to 9568 gC m^-2^, which is approximately twice as large as that in Korean rice paddy systems [32]. Conventional management of grassland soil includes application of compost and/or N fertilizer. Compost application generally increases soil CO_2_ evolution, while the application of N fertilizer is closely related to an increase in N_2_O emission [35–37]. We investigated changes in CO_2_ and N_2_O evolution from soil treated both with biochar and other amendments. Two different types of amendments (compost and urea) were chosen because these are commonly used in current grassland management in Korea. To the best of our knowledge, this is the first field site experiment in South Korea to investigate the effects of biochar on the soil ecosystem. We related changes in CO_2_ and N_2_O emissions to soil C and N dynamics and evaluated whether application of biochar is a sustainable management practice to simultaneously control greenhouse gas emissions and soil N availability.

## Materials and Methods

### Experimental site and basic physicochemical properties of biochar and soil

The field experiment was established on cultivated grassland located in Chunan-Si, Chungcheongnam-Do, Korea (East longitude 127°, North latitude 36°), where the average annual temperature and precipitation are 12.5°Cand 1226.5 mm, respectively (Korea Meteorological Administration).The field site was set up on Oct. 25, 2011 and the permission for the location was issued and managed by National Institute for Animal Science (NIAS), Korea. The mix-seeding rates were tall fescue (*Festuca arundinacea*) 8 kg ha^-1^, orchard grass (*Dactylis glomerata*) 15 kg ha^-1^, perennial ryegrass (*Lolium perenne*) 5 kg ha^-1^, and white clover (*Trifolium repens*) 2 kg ha^-1^.

The biochar used was a commercial product sold by the Farmers’ Association in Gangjin-gun, Korea. Biochar was produced by pyrolyzing rice husks at 500–600°Con a small-scale pyrolysis reactor (DCH-400, 1.4 m×5.2 m×5 m (L×W×H) from Daewon GSI Co., Korea.The amount of rice husk biochar processed was 400 kg h^-1^. The residence time was 110–120 min and the overall yield was 43%. The particle size of rice husks was 5mm or less before pyrolysis.

Biochar and soil pH were determined at a 1:5 ratio of air-dried biochar or soil to deionized water(w/v). Biochar surface area was measured by the BET method with N_2_ gas using mill-ground biochar. Soil texture was determined by a hydrometer and the cation exchange capacity (CEC) of soil was measured using an unbuffered salt extraction method [38].Total carbon (TC) content in the soil and biochar were analyzed by combustion analysis using a Carlo Erba NS 1500 C/N analyzer (Carlo Erba, Milan, Italy). NH_4_^+^-N and NO_3_^-^-N concentrations in the biochar and soil were determined through 2M potassium chloride (KCl) extraction and colorimetric methods[39].Metal content was analyzed by a spectroscopic method usingICP MS (Perkin-Elmer ICP-OES OPTIMA5300DV) to check whether the biochar contained metals that might be toxic to the soil ecosystem. The physicochemical properties of the biochar and soil are shown in Table 1.

**Table 1:** Physicochemical properties of the soil and biochar.

Soil														
Texture		pH	CEC		Bulk density		Total C		Total N	NH_4_^+^			NO_3_^-^	
			--- cmol kg^-1^ ---		------gcm^-3^-----		--------g kg^-1^ ------			--------- mg kg^-1^----------				
Sandy loam		7.20	1.57		1.27		4.23		0.58	10.22			30.13	
Biochar														
pH	Surface Area (BET)	Total C	Total N	HWC^*^	NH_4_^+^	NO_3_^-^	Al	Ca	Fe		Mg	K	Na	P
	---m^2^g^-1^---	---------- g kg^-1^ ------------			---mg kg^-1^---		--------------------------- g kg^-1^--------------------							
10.30	27.76	429.00	11.00	2.94	1.49	22.25	0.52	2.07	0.60		0.65	15.95	0.33	1.52

^*^HWC stands for hot water extractable C

### Experimental design and treatments

Treatments included biochar addition (NO CHAR, CHAR) and amendment (COMPOST, UREA, NO FERT). We used factorial design for six treatment combinations: NO CHAR/COMPOST, NO CHAR/UREA, NO CHAR/NO FERT, CHAR/COMPOST, CHAR/UREA, and CHAR/NO FERT. As we performed three replicates of each type, eighteen plots were completely randomized to the 22 m×37 m experimental field. The size of each plot was 5m×7m and individual plots were separated by protection rows that were 1m in width.

Biochar was applied once a year in fall after harvest (Oct. 25, 2011 and Sep. 21, 2012). The form of biochar applied was the particle of < 3 mm. The application rate of biochar was 0.3% by weight, which is equivalent to3.3 ton/ha when calculated based on a 10-cm depth field application. Biochar was incorporated into the soil profile down to a depth of 10 cm using shovels. The non-biochar amended plots were also mixed with a shovel. Soil amendment was conducted twice per year in fall and spring (Oct 25, 2011, March 21, 2012, Sep21, 2012, March 21, 2013). Amendment rates were140kg ha^-1^yr^-1^ of UREA and 1500 kg ha^-1^ yr^-1^of COMPOST, which are equivalent to 51.8 kg Nha^-1^yr^-1^and 11.3 kg Nha^-1^yr^-1^, respectively.

### Gas sampling and analysis

Gas samples were taken every month from 10/31/2011 to 05/01/2013 except over winter using a chamber method [29].Two chambers (20-cm diameter, 25-cm height) were inserted 5 cm deep into the soil for each plot. On sampling dates, the chambers were closed with airtight lids for 40 minutes and gas samples were withdrawn from the headspace of the closed chamber using a 10-ml three-way syringe (BD Luer-LokTip).

Gas samples were analyzed using a gas chromatograph (Agilent 7890A, USA) equipped with two detectors. CO_2_ was detected using a thermal conductivity detector (TCD) and N_2_O was detected using an electron capture detector (ECD).

Gas fluxes were calculated from the changes in headspace concentration over the measured period using the following equation [34]:

$$\text{Flux}=\frac{\text{dGas}}{\text{dt}}*\frac{\text{V}}{\text{A}}*\frac{\text{P}*100*\text{MW}}{\text{R}}*\frac{273}{273+\text{T}}$$ (1)

where, dGas/dt is the difference in gas concentrations between the initial and end time points, V is the volume of the chamber, A is the surface area which the chamber covers, P is the atmospheric pressure, MW is the molecular weight of the gas, R is a gas constant, 8314 J mol^-1^ K^-1^, and T is the absolute temperature.

### Soil sampling and analysis

On the same dates as gas sampling, soil samples were collected from each plot from a depth of 0–15 cm using a soil core sampler (4.9-cm i.d., Forest supplier, USA). Samples were sealed in marked plastic bags and taken to the laboratory after sampling.

For soil temperature measurements, a mini thermometer probe (Testo, 905-T1) was inserted to a depth of 10 cm. To measure soil gravimetric water content, approximately 15 g of soil was taken from each plastic bag and dried in the oven at 105°C for 24 h. Soil bulk density was determined by taking a soil core (4.9-cm diameter, 15-cm depth) and drying the soil at 105°C for 24 h to determine the soil dry weight contained in the known volume. Soil bulk density and gravimetric water content were used to determine the water-filled pore space (WFPS) of each soil core [39].

Temporal soil samples were passed through a 2-mm sieve and air-dried for 2 weeks before analysis. Labile organic C content of biochar was measured by the amount of hot water extractable C (HWC) following the method described in [40].To investigate N mineralization in soil, we measured NH_4_^+^-N and NO_3_^-^-N. Soil NH_4_^+^-N and NO_3_^-^-N concentrations were determined through 2M potassium chloride (KCl) extraction and colorimetric methods [41].Soil microbial activity was evaluated with the fluorescein diacetate (FDA) hydrolysis method [42].We analyzed the changes in soil pH, CEC, microbial biomass C and water holding capacity (WHC) for the last soil samples which was taken on 05/22/2013. Microbial analysis was measured by the CHCl_3_ fumigation extraction method [43, 44] and the WHC in the soil was determined using modification of the method described in [45].

To further examine the effect of biochar on soil N dynamics in the UREA soil, we set up a postulated balance of added urea N. The balance started with the amount of urea N applied which was 5180 mgN m^-2^. We assumed that 40% of applied urea N would be recovered in the plant biomass and soil organic N pool and this amount was not affected by biochar amendment [46]. We also assumed that 20% of applied urea were lost via NH_3_ volatilization process [47] and the amount of volatilization loss was not affected by biochar amendment. The remaining of the urea N was divided N_2_O emission, soil mineral N content (NH_4_^+^ + NO_3_^-^), and N loss from leaching. We calculated the amount of N leaching by subtracting measured N_2_O emission and soil mineral N content from the remaining amount of the urea N.

$$\text{Total amount of urea N}=plant\;biomass\;N+soil\;organic\;N+NH_{3}\;volatilization+N_{2}O\;emission+soil\;mineral\;N\;\left(NH_{3}^{+}+NO_{3}^{-}\right)+N\;leaching$$ (2)

### Statistical analyses

Analysis of variance (ANOVA) was performed using the MIXED procedure of SAS 9.2 [48] on CO_2_ emissions, N_2_O emissions, TC contents, bulk density, water filled pore space, microbial activity (FDA activity), HWC content, soil NH_4_^+^ and NO_3_^-^ contents. Biochar treatment, urea and compost amendment, and date were fixed effects. The ANOVA was performed separately on the soil pH, CEC, microbial biomass C, and WHC for the final soil sample and for this ANOVA, date was not considered as a fixed effect because the measurement was conducted only once. Least square means of the parameters were used to compare date, fertilization, and biochar effects. Pearson’s correlation coefficients among the CO_2_ emissions, N_2_O emissions, soil temperature, soil water content, and HWC content were calculated using the CORR procedure of SAS [48]. Statistical significance was set to the 5%probability level for all analyses.

## Results and Discussion

### Carbon dioxide emission and soil C dynamics

**Fig 1:**
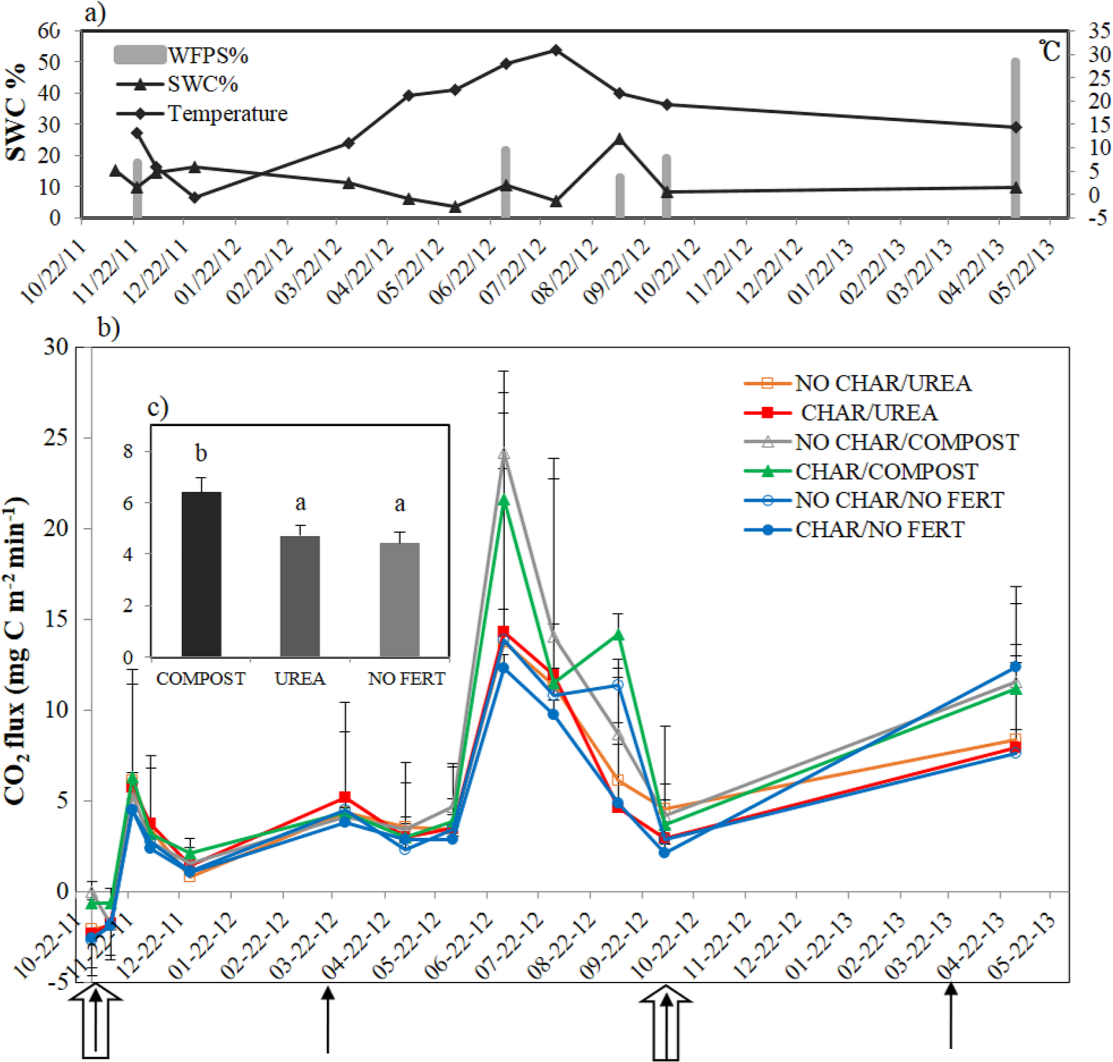
The temporal change of a) soil temperature, gravimetric soil water content, and water filled pore space (WFPS), b) CO_2_ evolution from the soil with biochar and amendments. c) Average soil CO_**2**_ emissions by different amendments. Four solid arrows show the urea and compost application events and two thick open arrows indicate the timing for biochar application. Error bars in c) represent the standard errors among the average data of the sampling dates.

The overall CO_2_emission pattern was positively correlated with soil temperature (Table 2, r = 0.610***), which has been reported by many researchers [22, 49, 50]. In contrast, the CO_2_ pattern was not correlated with soil water content, most likely because the gravimetric soil water content on our sampling dates was very low (range, 3.5–25.5%) (Fig 1A).

**Table 2:** Pearson correlation coefficients among CO_2_ emission rate, N_2_O emission rate, soil temperature, gravimetric water content, and hot water extractable C.

	CO_2_ emission rate	N_2_O emission rate	Soil Temperature	Soil water content	Hot water extractable C
CO_2_ emission rate	1	0.247* (0.0379)	0.610*** (<.0001)	0.124 (0.298)	0.586*** (<.0001)
N_2_O emission rate		1	0.216* (0.077)	-0.004 (0.975)	0.069 (0.568)
Soil Temperature			1	-0.060 (0.614)	0.626*** (<.0001)
Soil water content				1	-0.045 (0.710)
Hot water extractable C					1

Numbers in parentheses are the probability to reject the null hypothesis.

*, **, ***, significant at the P = 0.1, P = 0.05, P = 0.001 probability levels, respectively.

**Fig 2:**
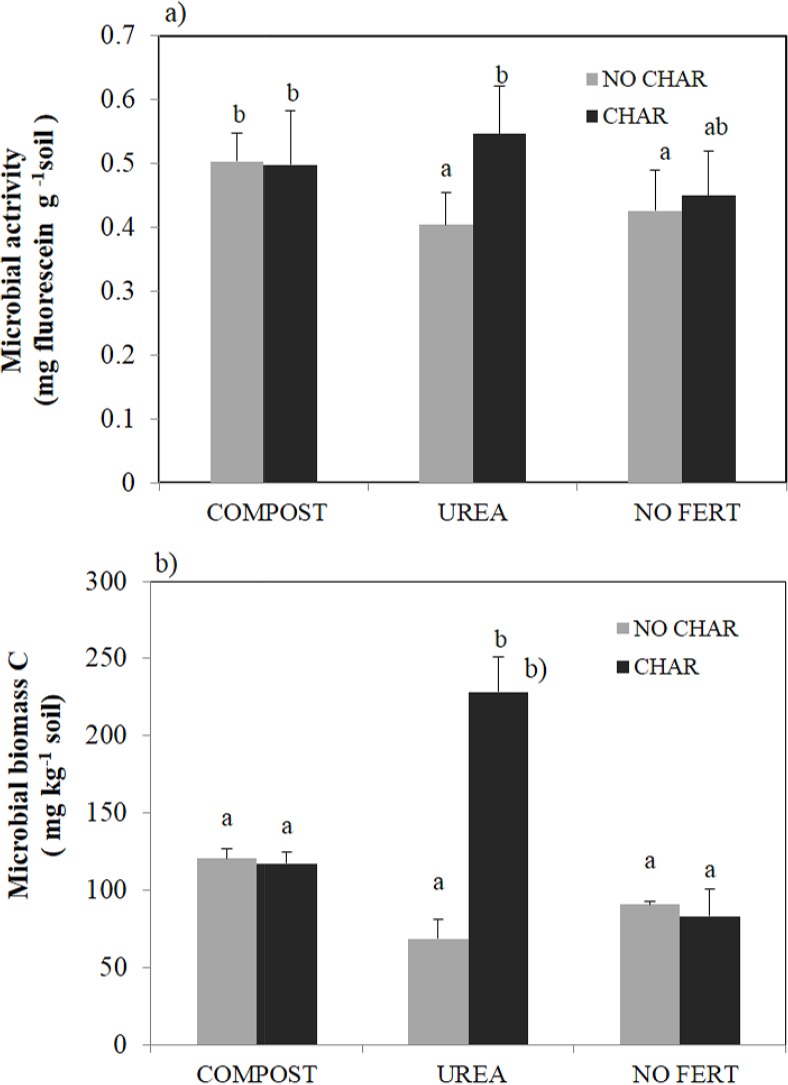
Interactive effect between biochar and amendments on a) microbial activity and b) microbial biomass C in the soil. Bars with different letters indicate significant differences in the average values of sampling dates among treatments at a 5% probably level.

An average throughout the 19 months of the field experiment revealed that overall CO_2_ emission from the COMPOST soil was significantly greater than that from the UREA and NO FERT soils (Table 3, Fig 1C). Greater CO_2_ emission from compost-treated soil than untreated soil has been reported previously by several researchers [51, 52]. Soluble C measured by hot water extractable C (HWC) from compost was hypothesized to stimulate the microbial community, resulting in higher CO_2_ evolution, and extractable organic C showed a high correlation with CO_2_ evolution (Table 3, r = 0.586***).However, unlike the consistent trend in HWC with CO_2_ emission, the patterns in FDA activity and MBC were not consistent with that of CO_2_ emission (Fig 2) and the correlations of them with CO_2_ emission rate were not significant. We attributed this inconsistency to the lower sensitivity of FDA activity and MBC to the treatment. It was widely reported that FDA activity and MBC are highly correlated [53] and they both represent “potential” microbial activity because FDA activity is measured after sufficient substrate is added and MBC includes dead biomass of microbes. Hence, we would say that “actual” microbial activity in the COMPOST soil might have been stimulated but it was not detected by these parameters.

**Table 3:** Results of analysis of variance by date, biochar and amendments.

Source	Soil analysis							Gas analysis	
	TC	BD	WFPS	FDA	NH_4_^+^	NO_3_^-^	HWC	CO_2_	N_2_O
	-----------------------------------------------------Pr > F------------------------------------------------------								
Date	<.0001***	<.0001***	<.0001***	<.0001***	<.0001**	<.0001***	<.0001***	<.0001***	<.0001***
CHAR	<.0001***	0.0421**	0.0826*	0.0506*	0.0004***	0.4625	0.0016***	0.4257	0.0714*
Date*CHAR	0.1771	0.3206	0.0001***	0.0455**	<.0001***	0.5901	0.0310**	0.5861	0.5008
AMEND*	0.0383**	0.0061***	0.0008***	0.1733	0.0049***	0.0021***	0.0026***	<.0001***	<.0001***
Date*AMNED	0.6267	0.864	0.0065***	0.6512	0.1040	0.0165**	0.0143**	<.0001***	<.0001***
CHAR*AMEND	0.0896*	0.8321	0.5562	0.0607*	0.0006***	0.6644	0.8550	0.5731	0.0332**
Date*CHAR*AMNED	0.7542	0.598	0.0473**	0.2287	0.0078***	0.9952	0.2813	0.0001***	0.0001***
Soil analysis									
Source	WHC			pH	CEC			MBC	
	-----------------------------------------Pr > F--------------------------------------------------								
CHAR	0.0449**			0.8768	0.5902			0.0006***	
AMEND	0.2686			0.4064	0.8457			0.0023***	
CHAR*AMEND	0.0900*			0.8010	0.5538			<.0001***	

*,**, ***, significant at the P = 0.1, P = 0.05, P = 0.001 probability levels, respectively.

*AMEND means application of the compost and urea; CHAR means biochar addition treatment; BD, soil bulk density; WFPS, soil water filled pore spaces; FDA, fluorescein diacetate hydrolysis activity; HWC, hot water extractable carbon; WHC, water holding capacity; CEC, cation exchange capacity; MBC, microbial biomass carbon.

The temporal pattern of CO_2_ emissions differed according to soil amendment type and biochar addition (Table 3II). Addition of biochar did not significantly affect CO_2_ emissions from any of the amended or control soils at the start of the experiment until 06/01/2012 (Table 3, Fig 1C). During Jul 2012, when the soil temperature and CO_2_ emission level were both very high, biochar addition had a negative effect onCO_2_ flux in the COMPOST soil (Fig 1B).From Aug 2012 to May 2013, we again did not observe any effects of biochar addition on CO_2_ emission from any of the soils. The reason why the biochar treatment only had an effect on CO_2_ emission when the temperature was high may in part be due to temporal changes in HWC (Table 4). The HWC content on 07/22/2012 was the highest measured, and on the same day, it was higher in the CHAR soils than in the NO CHAR soils. This result implies that labile C contained in the COMPOST soil was respired more freely in the NO CHAR soil than the CHAR soil. The suppression of CO_2_ emission by biochar was also reported in [19]; these authors attributed this to the high adsorptive affinity of biochar for existing organic C. We also argue that the higher content of labile C in the COMPOST soil protected from mineralization by biochar, resulting in a low CO_2_ evolution rate in this treatment.

**Table 4:** Seasonal change in the soil hot water extractable C (HWC) concentrations by biochar and amendments. Comparison was made within the column by biochar and amendments within one date.

Soil HWC concentration					
---------------------------------------g kg^-1^ soil----------------------------------------					
Source		03/29/2012	07/22//2012	10/28/2012	05/01/2013
COMPOST	NO CHAR	0.19a	0.33b	0.29a	0.24a
	CHAR	0.20a	0.49c	0.28a	0.21a
UREA	NO CHAR	0.20a	0.25a	0.22a	0.18a
	CHAR	0.21a	0.31b	0.27a	0.24a
NO FERT	NO CHAR	0.19a	0.28a	0.20a	0.18a
	CHAR	0.23a	0.36b	0.23a	0.21a

Values followed by the same letter are not significantly different at a 5% probability level.

**Fig 3:**
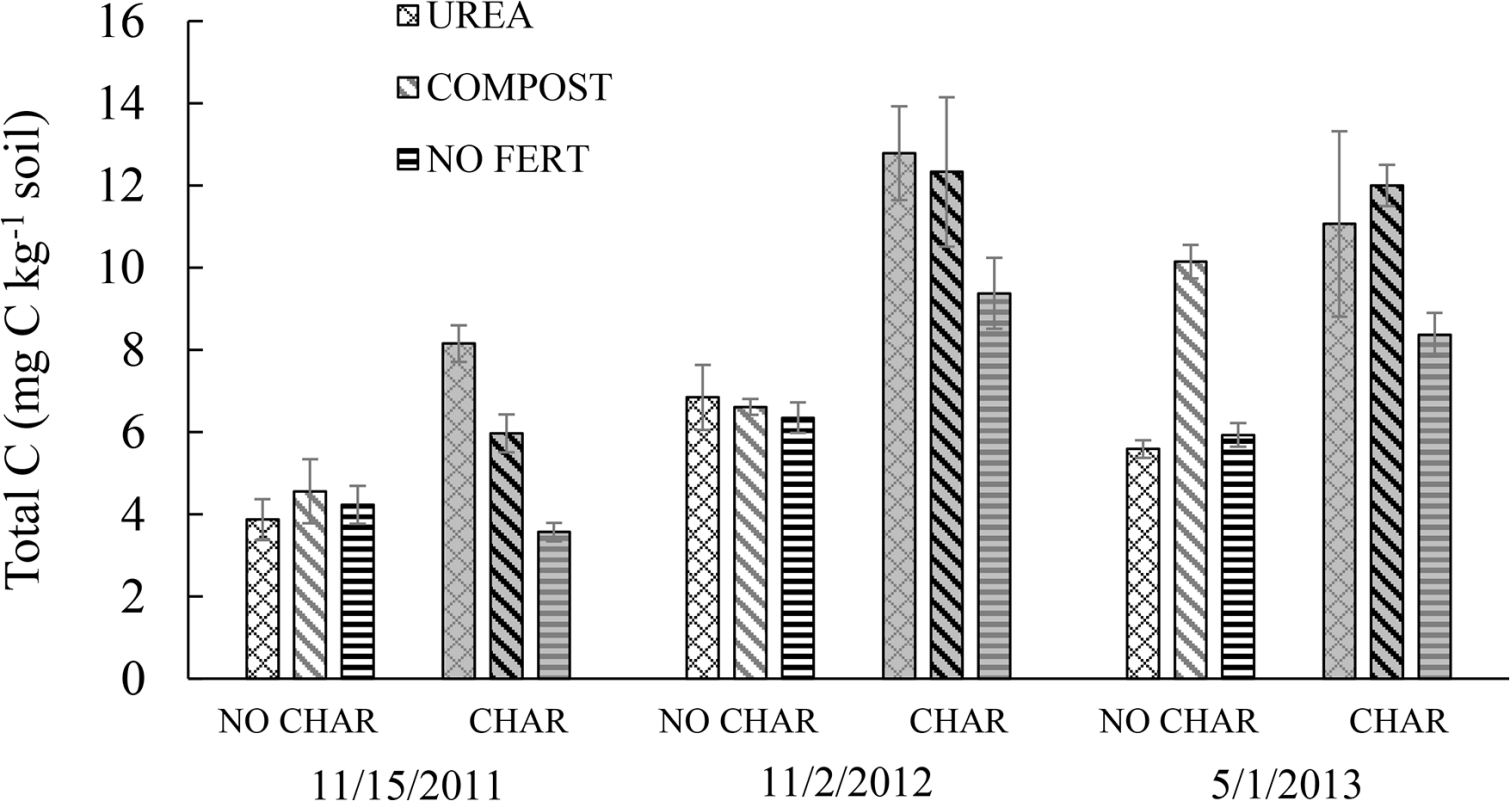
Temporal change in total C contents influenced by biochar and amendments. Bars with different letters indicate significant differences among treatments at a 5% probability level.

The pattern of soil CO_2_ emission was consistent with that of total C content (Fig 3). On average, soils with char addition had a 139.28% soil C content in the 19 months after biochar was first applied. In soils with biochar addition, total C was slightly higher in Nov 2012 than May 2013. The reason is that soil samples collected in Nov 2012 contained a lot of newly incorporated biochar particles, because we applied biochar in Sep 2012 for the second time, therefore there was not enough time for the biochar to become completely mixed with the soil. In May 2013, the soil C content was the highest in the COMPOST soil with biochar, and this result is consistent with low CO_2_ emission from the COMPOST soil amended with biochar during the hot summer days. Overall, the data imply that when biochar is applied together with compost, higher C sequestration can be expected, probably because decomposition of compost C is lowered by adsorption of labile C by biochar.

### Nitrous oxide emissions and soil available N dynamics

The overall patternofN_2_O emission showed a correlation with soil temperature (Table 2, r = 0.216*), but no correlation with soil water content. Soil water status has been reported to have a strong effectonN_2_O emission by several research groups [23, 39, 54, 55]. However, because the soil water content on our sampling dates was very low (less than 25% gravimetric water content) (Fig 1A), we did not observe a significant correlation between soil water content andN_2_O emission.

**Fig 4:**
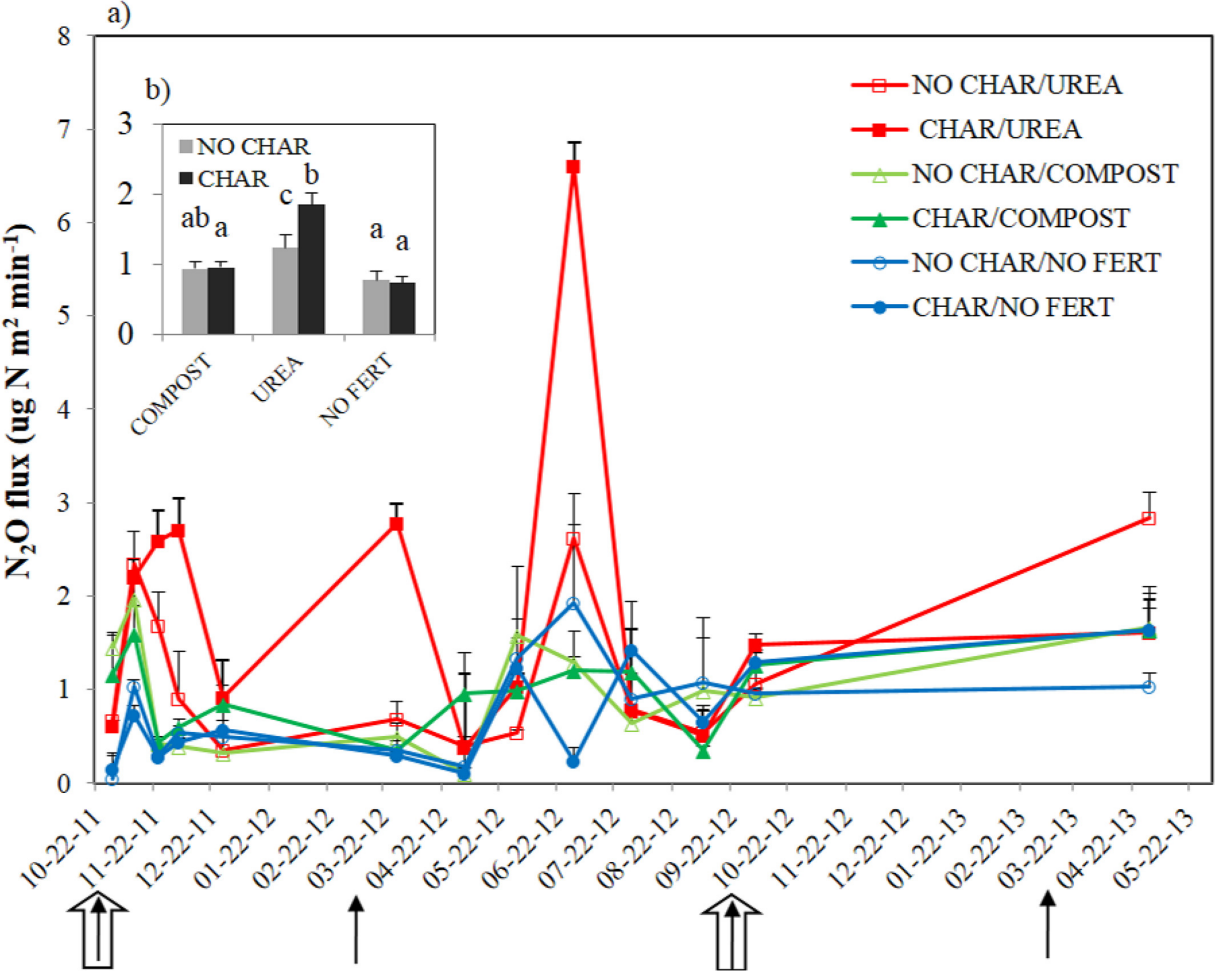
The N_2_O emissions represented as a) the temporal changes from the soils with biochar and amendments and b) the average of the interactive effects between biochar and amendments. Four solid arrows show the urea and compost application events and two thick open arrows indicate the timing for biochar application. Error bars in b) represent the standard errors among the average data of the sampling dates.

N_2_O emission was significantly influenced by the interaction between amendment and biochar treatment (Fig 4A and 4B). In the COMPOST and NO FERT soils, biochar amendment did not change N_2_O emission at any point during the experiment. In contrast, biochar addition to UREA soil significantly stimulated N_2_O emission by 49% on average compared to soil without biochar. This result is consistent with the results reported in [56], namely thatN_2_O emission was significantly increased by biochar addition, especially when added together with mineral fertilizer N. Other researchers have also reported that biochar treatment of fertilized soil results in higher N_2_O emission than from the fertilized soil alone [32, 39, 57]. However, contrary to our results, it was reported that biochar treatment significantly reduced N_2_O emission from rice paddy soils with no N fertilization [19, 29]. In the mechanism-based research of [29], 11 of 15 agricultural soils were found to emit a lower level of N_2_O upon biochar amendment, and these authors discussed the interaction between black C and N dynamics. However, no change in N_2_O emission in response to biochar addition was also noted when urea N was added [20]. These inconsistent results indicate that the effect of biochar on soil N_2_O emission is dependent on water status, pH, C status, and N fertilizer management of soils [4, 33, 58].

**Fig 5:**
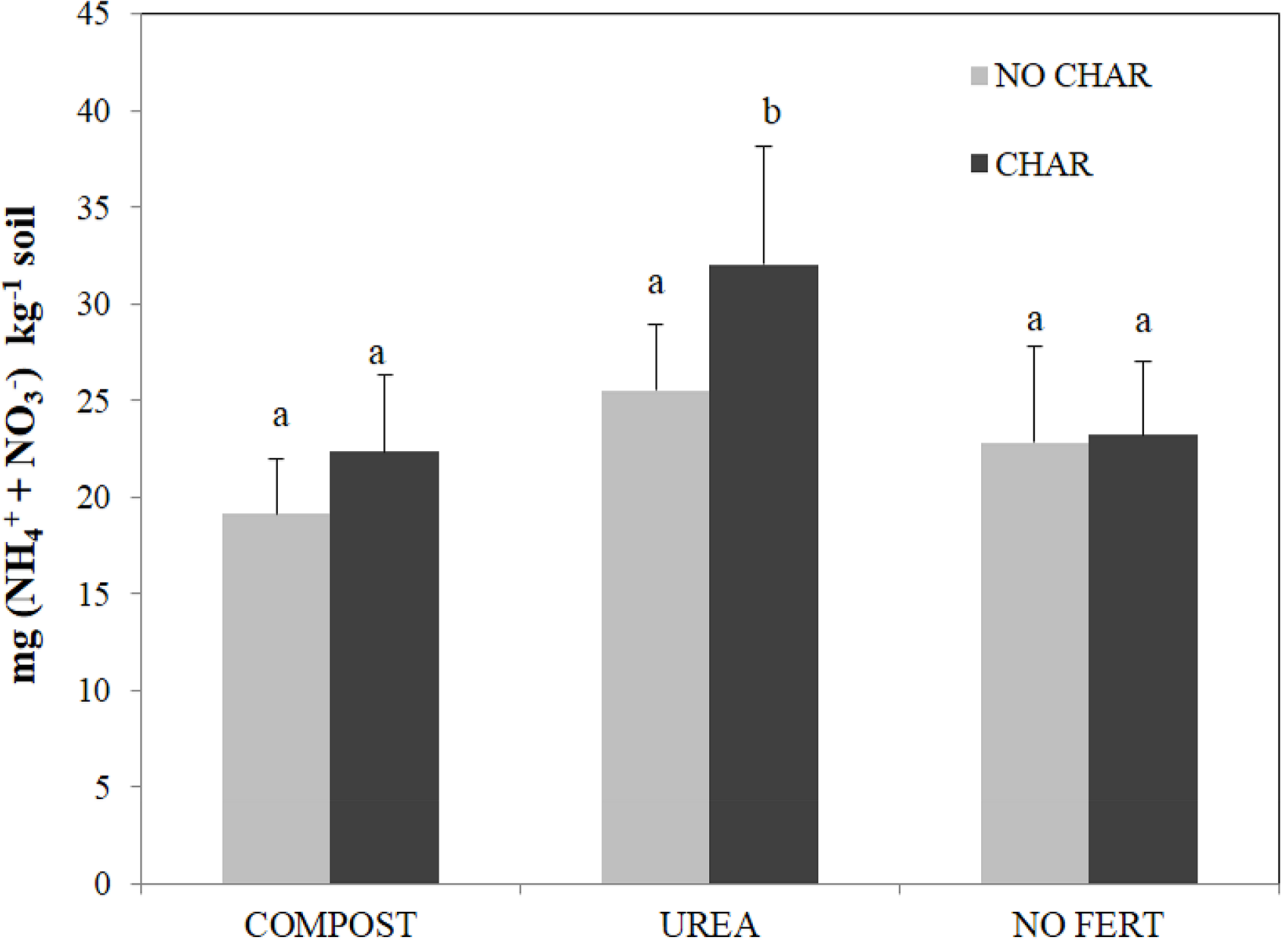
Effects of biochar and amendments on the average amounts of NH_4_^+^ + NO_3_^-^.Bars with different letters indicate significant differences among treatments at a 5% probability level.

In our study, we assumed that most of the N_2_O produced was from autotrophic and heterotrophic nitrification, because our WFPS ranged from 7–57%, which we assumed meant that the soil was aerobic. It has been reported that all of N_2_O emitted over 70% WFPS was produced during denitrification, but that at 35–60% WFPS, nitrification was the main process producing N_2_O [26]. Apart from the autotrophic nitrification process, heterotrophic nitrification has also been reported to be an important mechanism to remove NH_4_^+^ under aerobic conditions [59]. If we assume that heterotrophic nitrification is the main process resulting in N_2_O emission from the UREA soil, the higher N_2_O emission resulting from CHAR treatment of this soil can be explained as follows. In the CHAR treatment of UREA soil, consistently higher amounts of HWC and mineral N were observed (Table 4, Fig 5). Hence, the combination of a higher amount of labile C and mineral N can explain the higher rate of N_2_O emission from UREA soil with CHAR treatment compared to UREA soil with NO CHAR treatment due to stimulation of heterotrophic nitrification. This interaction was especially important at our field site because our soil was severely C limited due to a very low organic C content (0.4% weight basis). We did not consider biochar-derived mineral N as making a direct contribution to the higher mineral N content in the UREA soil because the mineral N content in the biochar has been reported to be very low [60] and was not increased by biochar addition to the COMPOST and NO FERT soils. This argument is further supported by the microbial FDA activity and microbial biomass C results (Fig 2). Higher FDA activity and microbial biomass C in the UREA soil with biochar addition indirectly indicated higher microbial autotrophic and heterotrophic nitrification in this soil.

**Fig 6:**
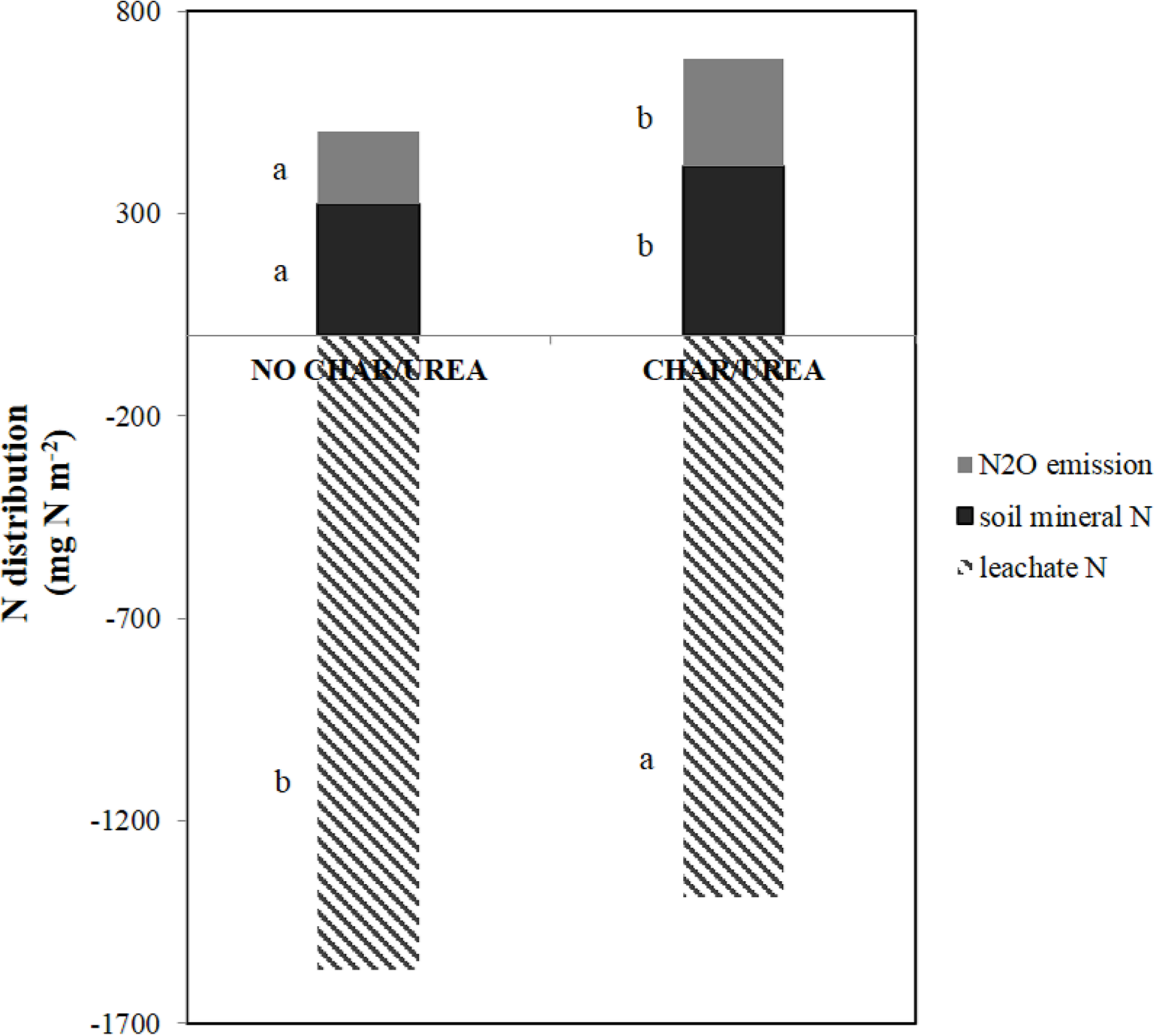
Postulated N balance in the UREA soil with and without biochar addition. Total amounts of urea N in the soil were assumed to be lost through N_**2**_O emission, NH_**3**_ volatilization and leaching and be remained in the soil as organic N and mineral N (NH_**4**_^**+**^ + NO_**3**_^**-**^). The amount of NH_**3**_ volatilization, plant biomass N and soil organic N were assumed to be the same between NO CHAR and CHAR treatments and not shown in the graph. Different letters beside the bars indicate significant differences between the NO CHAR and CHAR treatments at a 5% probability level.

**Fig 7:**
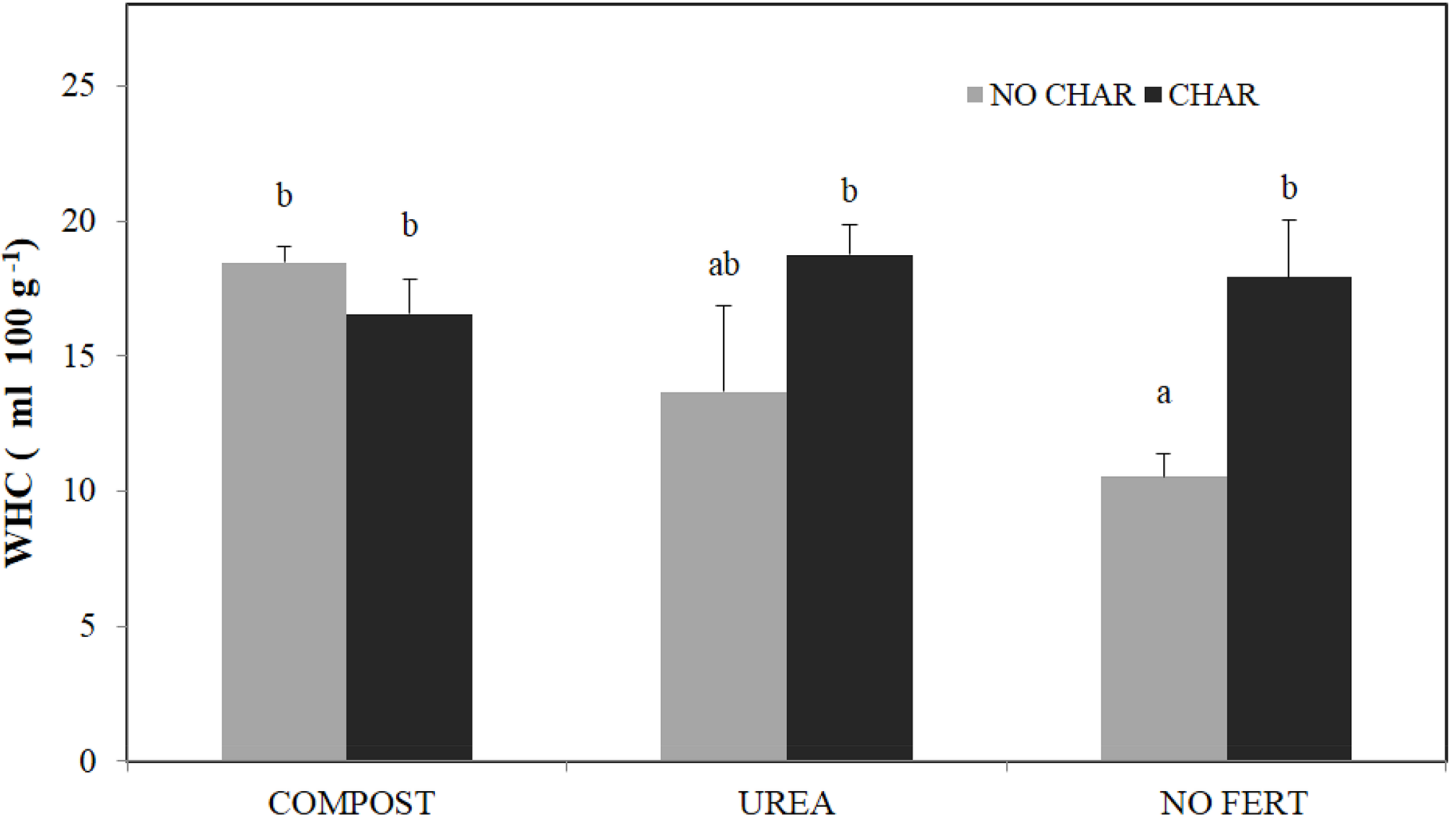
Effect of biochar and fertilization on soil water holding capacity (WHC).Bars with different letters indicate significant differences among treatments at a 5% probability level.

Another possible mechanism to explain the higher N_2_O emission from the soil amended with both urea and biochar is as follows. Theoretical distribution of urea-derived mineral N in the UREA soil revealed that in the biochar-amended soil, N loss through N_2_O emission was higher by 48.89% and the soil mineral N content was higher by 28.13% than in soil without biochar. In contrast, N loss via leaching was significantly lower in CHAR soil than in the NO CHAR soil (Fig 6). This result is consistent with [61], which observed that soil with biochar addition showed less NO_3_^-^ leaching than soil without biochar. They proposed that the soil with biochar had better water-holding capacity due to the enhanced volume provided by soil mesoaggregates. In this study, we also observed higher WHC in the UREA and NO FERT soils with biochar addition (Fig 7), further supporting that N loss through leaching is reduced in biochar-treated soil.

In summary, C-poor soil treated with urea fertilizer and biochar was able to retain a higher amount of mineral N than untreated soil. This could supply sufficient available N for plant growth and explain the reported increases in plant yield in response to biochar addition [62, 63]. However, the higher retainment of mineral N within the soil might cause greater N loss through N_2_O emissions, which is a strong greenhouse gas. Therefore, when applying biochar and urea to C-poor soils, the growth pattern of plants should be considered to determine the best timing to maximize the plant’s utilization and minimize N_2_O loss.

## Conclusions

As grassland ecosystems have the potential to sequester significant amounts of C, development of good management practices for these systems is needed to enhance their potential to mitigate climate change by maximizing C sequestration and minimizing greenhouse gas emissions. When both compost and biochar were applied to soil, less CO_2_evolved from the soil during the summer, probably due to adsorption of labile C by the biochar. Soil C sequestration was highest in the COMPOST soil with biochar addition, consistent with the pattern of CO_2_ evolution.

Soil N_2_O emissions were significantly influenced by interactions between the biochar and amendment materials. Higher N_2_O emission was observed in UREA soil with biochar than without it, while in the COMPOST and NO FERT soils, there was no difference in N_2_O emissions between soils with and without biochar. We proposed two possible mechanisms to explain the higher N_2_O emission from UREA soil amended with biochar. The first mechanism was enhanced autotrophic and heterotrophic nitrification due to interaction between labile C from the biochar and mineral N. When soil is C limited, as was the soil in our study, the addition of biochar could provide the soil with labile C, resulting in enhanced N mineralization (ammonification) and heterotrophic nitrification, finally leading to an increase in N_2_O emission. The second mechanism was enhanced retention of mineral N content by the biochar. Mineral N could easily be leached out if there was no holding mechanism in the soil. Biochar addition reduced N loss due to leaching in the UREA soil, leading to greater retention of available N, which resulted in higher N_2_O emissions from the soil. The overall N_2_O emission results indicate that care should be taken when applying biochar together with urea fertilizer. To develop a sustainable management strategy for fertilizer and biochar application, the timing of N fertilizer and biochar application should take into consideration of N loss through both N_2_O emission and leaching. In future studies, we intend to trace fertilizer N using an isotope to gain a mechanistic understanding of the effects of biochar on soil N dynamics.

## References

[1] Lehmann J, Gaunt J, Rondon M (2006) Bio-char sequestration in terrestrial ecosystems—a review. Mitig Adapt Strat Gl 11: 395–419.

[2] Laird D, Fleming P, Wang B, Horton R, Karlen D (2010) Biochar impact on nutrient leaching from a Midwestern agricultural soil. Geoderma 158: 436–442.

[3] Zhang A, Bian R, Pan G, Cui L, Hussain Q, Li L, et al (2012a) Effects of biochar amendment on soil quality, crop yield and greenhouse gas emission in a Chinese rice paddy: A field study of 2 consecutive rice growing cycles. Field Crop Res 127: 153–160.

[4] Chintala R, Mollinedo J, Schumacher TE, Malo DD, Papiernik SK, Malo DD, et al (2013) Nitrate sorption and desorption in biochars from fast pyrolysis. Microporous and Mesoporous Materials 179: 250–257.

[5] Chintala R, Schumacher TE, McDonald LM, Clay DE, Malo DD, Papiernik SA, et al (2013) Phosphorus sorption and availability from biochars and soil/biochar mixtures. CLEAN-Soil Air Water 41:626–634.

[6] Spokas A, Reicosky DC (2009) Impacts of sixteen different biochars on soil greenhouse gas production. Annals of Environmental Science 3: Article 4.

[7] Van Zwieten L, Kimber S, Downie A, Morris S, Petty S, Rust J, et al (2010) A glasshouse study on the interaction of low mineral ash biochar with nitrogen in a sandy soil. Soil Res 48: 569–576.

[8] Bruun EW, Ambus P, Egsgaard H, Hauggaard-Nielsen H (2012) Effects of slow and fast pyrolysis biochar on soil C and N turnover dynamics. Soil Biol Biochem 46: 73–79.

[9] Chintala R, Schumacher TE, Kumar S, Malo DD, Rice JA. (2014) Molecular characterization of biochar materials and their influence on microbiological properties of soil. J Hazard Materials 279:244–256. 10.1016/j.jhazmat.2014.06.074 25064262

[10] Liang B, Lehmann J, Solomon D, Kinyangi J, Grossman J, O'Neill B, et al (2006) Black carbon increases cation exchange capacity in soils. Soil Sci Soc Am J 70: 1719–1730.

[11] Ogawa M (1994) Symbiosis of people and nature in the tropics. Farming Japan. 28:10–34.

[12] Steiner C, Das KC, Garcia M, Forseter B, Zech W (2008) Charcoal and smoke extract stimualte the soil microbial community in a highly weathered xanthinc Ferralsol. Pedobiologia 51:359–366.

[13] DeLuca TH, Sala A (2006) Frequent fire alters nitrogen transformations in pondersoa pine stands of the inland northwest. Ecology 87:2511–2522. 17089660 10.1890/0012-9658(2006)87[2511:ffanti]2.0.co;2

[14] Lehmann J, Kern DC, Glaser B, Woods WI (2003) Amazonian dark earths: origin, properties, management Dordrecht: Kluwer academic publishers.

[15] Cross A, Sohi SP (2011) The priming potential of biochar products in relation to labile carbon contents and soil organic matter status Soil Biol. Biochem 43: 2127–2134.

[16] Jones DL, Rousk J, Edwards-Jones G, DeLuca TH, Murphy DV (2012) Biochar-mediated changes in soil quality and plant growth in a three year field trial. Soil Biol Biochem 45: 113–124.

[17] Ameloot N, De Neve S, Jegajeevagan K, Yildiz G, Buchan D, Funkuin YN, et al (2013) Short-term CO_2_ and N_2_O emissions and microbial properties of biochar amended sandy loam soils. Soil Biol Biochem 57: 401–410.

[18] Zimmerman AR, Gao B, Ahn MY (2011) Positive and negative carbon mineralization priming effects among a variety of biochar-amended soils. Soil Biol Biochem 43: 1169–1179.

[19] Wang J, Pan X, Liu Y, Zhang X, Xiong Z (2012) Effects of biochar amendment in two soils on greenhouse gas emissions and crop production. Plant Soil 360: 287–298.

[20] Awad YM, Blagodatskaya E, Ok YS, Kuzyakov YY (2012) Effects of polyacrylamide, biopolymer, and biochar on decomposition of soil organic matter and plant residues as determined by 14C and enzyme activities. Eur J Soil Biol 48:1–10.

[21] Ahmad M, Lee SS, Dou X, Mohan D, Sung JK, Yang JE, et al (2012) Effects of pyrolysis temperature on soybean stover and peanut shell-derived biochar properties and TCE adsorption in water. Bioresource Technology 118:536–544. 10.1016/j.biortech.2012.05.042 22721877

[22] Case SDC, McNamara NP, Reay DS, Whitaker J (2012) The effect of biochar addition on N_2_O and CO_2_ emissions from a sandy loam soil—The role of soil aeration. Soil Biol Biochem. 51: 125–134.

[23] Chintala R, Owen RK, Schumacher TE, Spokas KA, McDonald LM, D.D Malo, et al. (2014) Denitrification kinetics in biomass and biochar amended soils of different landscape positions. Environ Sci Pollut Res 10.1007/s11356-014-3762-2 25369917

[24] Castingnetti D, Hollocher T (1984) Heterotrophic nitrification among denitrifiers. Appl Environ Microbiol 47: 620–623 6721486 10.1128/aem.47.4.620-623.1984 PMC239737

[25] Wragea N, Velthofb GL, Van Beusichema ML, Oenema O (2001) Role of nitrifier denitrification in the production of nitrous oxide. Soil Biol Biochem 33: 1723–1732.

[26] Bateman EJ, Baggs EM (2005) Contributions of nitrification and denitrification to N_2_O emissions from soils at different water-filled pore space. Biol Fertil Soils. 41: 379–388.

[27] Rondon MA., Molina D, Hurtado M, Ramirez J, Lehmann J, Major J,et al. (2006) Enhancing the productivity of crops and grasses while reducing greenhouse gas emissions through bio-char amendments to unfertile tropical soils. In: 18th World Congress of soil science, Philadelphia, PA, 9–14 July 2006. http://crops.confex.com/crops/wc2006/techprogram/P16849.HTM, Accessed June 2008

[28] Hayatsu M, Tago K, Saito M (2008) Various players in the nitrogen cycle: diversity and functions of the microorganisms involved in nitrification and denitrification. Soil Sci Plant Nutr 54: 33–45.

[29] Wang J, Zhang M, Xiong Z, Liu P, Pan G (2011) Effects of biochar addition on N_2_O and CO_2_ emissions from two paddy soils. Biol Fert Soils 47: 887–896.

[30] Zhang A, Liu Y, Pan G, Hussain Q, Li L, Zheng J, Zhang X (2012b) Effect of biochar amendment on maize yield and greenhouse gas emissions from a soil organic carbon poor calcareous loamy soil from Central China Plain. Plant Soil 351: 263–275.

[31] Rogovska N, Laird D, Cruse R, Fleming P, Parkin T, Meek D (2011) Impact of biochar on manure carbon stabilization and greenhouse gas emissions. Soil Sci Soc Am J 75: 871–879.

[32] Yoo G, Kang H (2012) Effects of biochar addition on greenhouse gas emissions and microbial responses in a short-term laboratory experiment. J Environ Qual 41: 1193–1202. 10.2134/jeq2011.0157 22751062

[33] Cayuela ML, Sánchez-Monedero MA, Roig A, Hanley K, Enders A, Lehmann J (2013) Biochar and denitrification in soils: when, how much and why does biochar reduce N_2_O emissions? Sci Rep 3: Article 1732. 10.1038/srep01732 PMC3635057 23615819

[34] Troy SM, Lawlor PG, O'Flynn CJ, Healy MG (2013) Impact of biochar addition to soil on greenhouse gas emissions following pig manure application. Soil Biol Biochem 60: 173–181.

[35] Bouwman AF (1996) Direct emission of nitrous oxide from agricultural soils. Nutrient Cycling in Agroecosystems 46:53–70.

[36] Burton DL, Li X, Grat C A (2008) Influence of fertilizer nitrogen source and management practice on N_2_O emissions from two Black Chernozemic soils. Can J Soil Sci 88:219–227.

[37] Gao X, Tenuta M, Nelson A, Sparling B, Tomasiewicz D, Mohr RM, et al (2013) Effect of nitrogen fertilizer rate on nitrous oxide emission from irrigated potato on a clay loam soil in Manitoba, Cananda Can J Soil Sci 93:1–11.

[38] Sparks DL, Page A, Helmke P, Loeppert R, Soltanpour P, Tabatabai M, et al. (1996) Methods of soil analysis. Part 3 Chemical methods. Soil Science Society of America Book Series, No 5.

[39] Yanai Y, Toyota K, Okazaki M (2007) Effects of charcoal addition on N_2_O emissions from soil resulting from rewetting air-dried soil in short-term laboratory experiments. Soil Sci Plant Nutr 53: 181–188

[40] Haynes RJ, Francis GS(1993) Changes in microbial biomass C, soil carbohydrate composition and aggregate stability induced by growth of selected crop and forage species under field conditions. Eur J Soil Sci 44: 665–675.

[41] Sims GK, Ellsworth TR, Mulvaney RL (1995) Microscale determination of inorganic nitrogen in water and soil extracts. Commun Soil Sci Plan 26: 303–316.

[42] Adam G, Duncan H (2001) Development of a sensitive and rapid method for the measurement of total microbial activity using fluorescein diacetate (FDA) in a range of soils. Soil Biol Biochem 33: 943–951.

[43] Vance ED, Brookes PC, Jenkinson DS (1987) An extraction method for measuring soil microbial biomass C. Soil Biol Biochem 19: 703–707.

[44] Beck T, Joergensen RG, Kandeler E, Makeschin F, Nuss E, Oberholzer HR, Scheu S (1997) An inter-laboratory comparison of ten different ways of measuring soil microbial biomass C. Soil Biol Biochem 29: 1023–1032.

[45] Pramer D, Schmidt EL (1965) Experimental Soil Microbiology. Minneapolis MN: Burgess

[46] Gava GJC, Trivelin PCO, Vitti AC, Oliveira MW de (2005) Urea and sugarcane straw nitrogen balance in a soil-sugarcane crop system. Pesq Agropec Bras Brasilia 40:689–395.

[47] Ellington A, (1986) Ammonia volatilization losses from fertilizers applied to acid soil in the field. Fert Res 8: 283–296

[48] Institute SAS (2008) SAS User’s guide SAS Inst Cary, NC

[49] Keith H, Jacobsen K L, Raison RJ (1997) Effects of soil phosphorus availability, temperature and moisture on soil respiration in Eucalyptus pauciflora forest. Plant Soil 190: 127–141

[50] Karhu K, Mattila T, Bergstrom I, Regina K (2011) Biochar addition to agricultural soil increased CH_4_ uptake and water holding capacity-Results from a short-term pilot field study. Agr Ecosyst Environ 140:309–313.

[51] Rochette P, Gregorich EG (1998) Dynamics of soil microbial biomass C, soluble organic C and CO_2_ evolution after three years of manure application. Can J Soil Sci 78:283–290.

[52] Wu L, Ma LQ (2002) Relationship between compost stability and extractable oragnic carbon. J Environ Qual 31:1323–1328. 12175053 10.2134/jeq2002.1323

[53] Sánchez-Monedero M A, Mondini C, Cayuela M L, Roig A, Contin M, De Nobili M. (2008) Fluorescein diacetate hydrolysis, respiration and microbial biomass in freshly amended soils. Biol.Fertil. Soils, 44:885–890

[54] Maag M, Vinther FP (1996) Nitrous oxide emission by nitrification and denitrification in different soil types and at different soil moisture contents and temperatures. Appl Soil Ecol 4: 5–14.

[55] Schindlbacher A, Zechmeister-Boltenstern S, Butterbach-Bahl K (2004) Effects of soil moisture and temperature on NO, NO_2_, and N_2_O emissions from European forest soils. J Geophys Res 109: D17302.

[56] Kammann C, Ratering S, Eckhard C, Müller C (2012) Biochar and hydrochar effects on greenhouse gas (carbon dioxide, nitrous oxide, and methane) fluxes from soils.J Environ Qual 41: 1052–1066. 10.2134/jeq2011.0132 22751047

[57] Clough TJ, Bertram JE, Ray J, Condron LM, O'Callaghan M, Sherlock RR, Wells N (2010) Unweathered wood biochar impact on nitrous oxide emissions from a bovine-urine-amended pasture soil. Soil Sci Soc Am J 74: 852–860.

[58] Clough TJ, Condron LM (2010) Biochar and the Nitrogen Cycle: Introduction.J Environ Qual 39: 1218–1223. 20830909 10.2134/jeq2010.0204

[59] Zhang JB, Wu PX, Hao B, Yu ZN (2011) Heterotrophic nitrification and aerobic denitrification by the bacterium Pseudomonas stutzeri YZN-001. Bioresour Technol 102:9866–9869. 10.1016/j.biortech.2011.07.118 21911288

[60] Chan KY, Xu ZH (2010) Biochar: nutrient properties and their enhancement In: Joseph S, Lehmann J, editors. biochar for environmental management science and technology. Gateshead: earthscan pp. 67–84

[61] Yoo GY, Kim HJ, Chen JJ, Kim YS (2014) Effects of Biochar Addition on Nitrogen Leaching and Soil Structure following Fertilizer Application to Rice Paddy Soil. Soil Sci Soc Am J 78: 852–860

[62] Major J, Rondon M, Molina D, Riha SJ, Lehmann J (2010) Maize yield and nutrition during 4 years after biochar application to a Colombian savanna oxisol. Plant Soil 333: 117–128.

[63] Alberquerque JA, Salazar P, Barron V, Torrent J, Campillo MC, Gallardo A, Vilar R (2013) Enhanced wheat yield by biochar addition under different mineral fertilization levels. Agron Sustai Dev 33: 475–484

